# A dataset of blockade, vandalism, and harassment activities for the cause of climate change mitigation

**DOI:** 10.1016/j.dib.2024.110342

**Published:** 2024-03-19

**Authors:** Quan-Hoang Vuong, Minh-Hoang Nguyen, Viet-Phuong La

**Affiliations:** a Centre for Interdisciplinary Social Research, Phenikaa University, Yen Nghia Ward, Ha Dong District, Hanoi, Viet Nam; b A.I. for Social Data Lab (AISDL), Vuong & Associates, Hanoi, Viet Nam

**Keywords:** Environmental activism, Art vandalism, Harassment, Road blockage, Climate change, Violence, Event disruption, Mindsponge theory

## Abstract

Environmental activism is crucial for raising public awareness and support toward addressing the climate crisis. However, using climate change mitigation as the cause for blockade, vandalism, and harassment activities might be counterproductive and risk causing negative repercussions and declining public support. The paper describes a dataset of metadata of 89 blockade, vandalism, and harassment events happening 13 countries in recent years. The dataset comprises three main categories: 1) Events, 2) Activists, and 3) Consequences. For researchers interested in environmental activism, climate change, and sustainability, the dataset is helpful in studying the effectiveness and appropriateness of strategies to raise public awareness and support. For researchers in the field of security studies and green criminology, the dataset offers resources to study features and impacts of blockade, vandalism, and harassment events. The Bayesian Mindsponge Framework (BMF) analytics was employed to validate the dataset. Consequently, the estimated result aligns with the Mindsponge Theory's theoretical reasoning.

Specifications Table

**Table:** 

Subject	Sociology; Management, Monitoring, Policy and Law; Criminology
Specific subject area	• Environmental activism • Environmental management • Green criminology
Type of data	Table Raw, Processed
Data collection	Initially, we found popular activism events in the mass media and identify the environmental groups frequently associated with those events. We then traced relevant information about these groups and gathered their conducted events, which are shown in the press. The information (i.e., news) collected from those events was later used to retrieve the metadata of the blockade, vandalism, and harassment events. In the second step, we designed a questionnaire to retrieve the metadata from the collected news for each event. The Mindsponge Theory was employed to design the structure of the questionnaire.
Data source location	News media The data is stored at Phenikaa University, Yen Nghia Ward, Ha Dong District, Hanoi, Vietnam.
Data accessibility	Repository name: Zenodo Data identification number: DOI:10.5281/zenodo.10811334 Direct URL to data: https://zenodo.org/records/10811334 Instructions for accessing these data: The dataset can be accessed and downloaded directly by using the provided URL. In case the link does not work, the dataset is deposited as a Supplementary to this data descriptor.

[...] only by uniting the power of the entire village could they chase Snake away. In “Virtue of Sacrifice”; The Kingfisher Story Collection [1].

## 1 Value of the Data

• The dataset offers resources to study blockade, vandalism, and harassment events that are claimed to be the cause of fighting climate change. The resources have been validated by the Bayesian Mindsponge Framework (BMF) analytics.
• Researchers in the field of environmental activism, climate change, and sustainability can use the dataset to generate knowledge about the effectiveness and appropriateness of strategies to raise public awareness and support.
• Researchers in the field of green criminology and security studies can benefit from analyzing the features and impacts of blockade, vandalism, and harassment events.
• Policymakers can use the dataset to evaluate the potential consequences of radical environmental movements for planning and implementing more appropriate responses.
• Making the data open reduces the cost of doing research on radical environmental activism, improves the study's integrity and transparency, and enhances research reproducibility.
• The dataset was generated from news reports on the events, so the accuracy of the observations is subject to bias caused by the news's incompleteness. Future data collection should be conducted through on-the-scene observations or official reports from the activists and police officers to validate the current dataset.

## 2 Background

Environmental activism is essential for increasing awareness about environmental deterioration and motivating people to reduce or halt ecologically detrimental actions, like those that cause climate change and biodiversity loss crises [2], [3], [4], [5]. Although environmental activists' enthusiasm and devotion should be recognized, various groups of activists are supporting the radical environmentalist movement that favors employing vandalism measures to achieve their principal purpose [6]. Such measures include but are not limited to the vandalism of priceless artworks by world-renowned painters, road blockades during rush-hour traffic, and harassment of business owners and managers [7,8]. Vandalism and harassment actions for promoting climate change mitigation can result in negative repercussions and may decline public support for the environmental cause.

More empirical studies are required to understand the impacts of inappropriate blockade, vandalism, and harassment actions. Therefore, we have compiled a dataset of blockade, vandalism, and harassment events that were popular in the mass media (e.g., news and social media posts) to support the study of these events. We expect the dataset will aid the knowledge generation in the field of environmental activism and societal transitions to adapt to climate change and reduce the cost of doing research [9], [10], [11], [12].

## 3 Data Description

### 3.1 Data sample

**Fig. 1:**
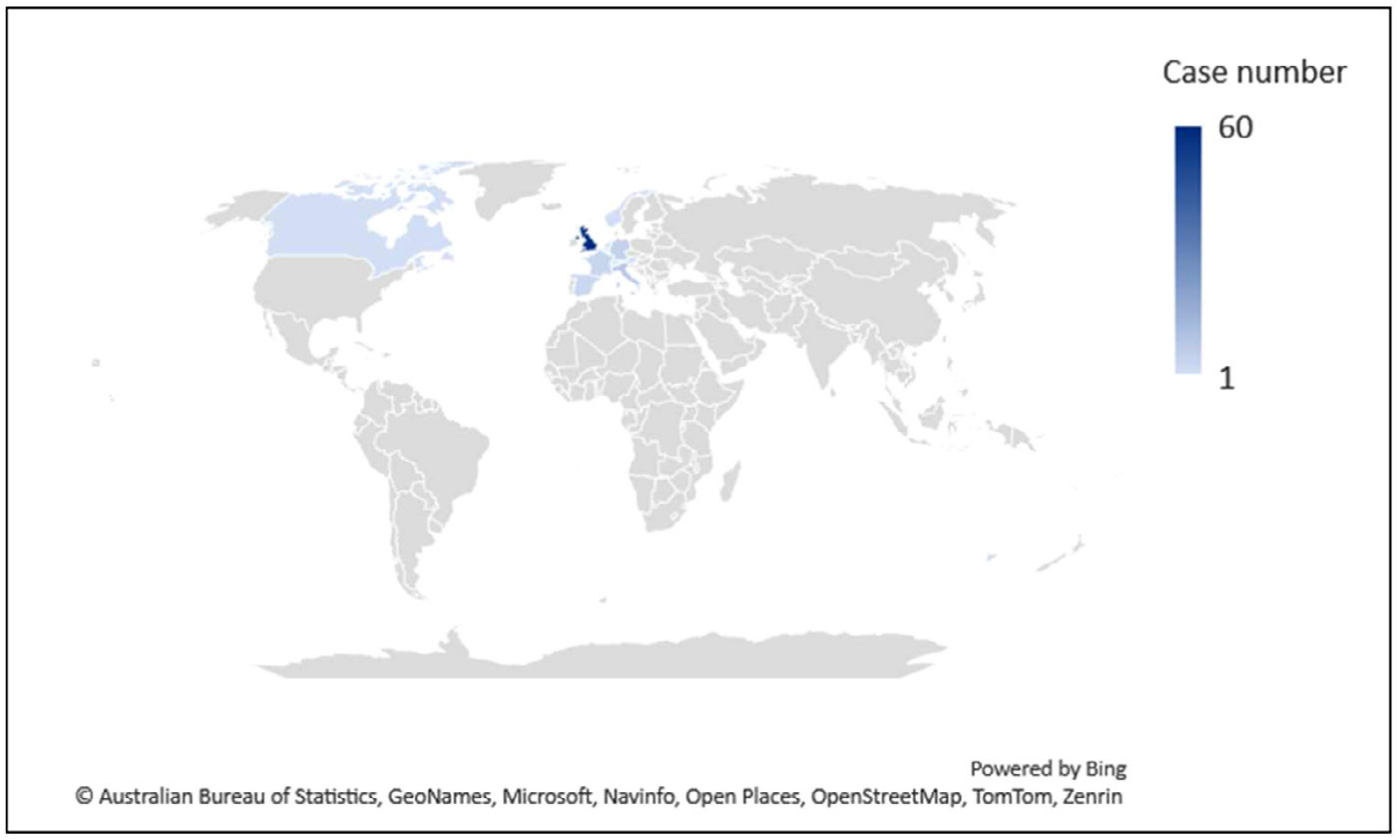
The distribution map of the blockade, vandalism, and harassment cases.

The dataset recorded 89 cases of blockade, vandalism, and harassment for the cause of fighting climate change that happened in 13 countries. These countries are primarily high-income Western countries in Europe; only Australia and Canada are two non-European countries (see Fig. 1). The United Kingdom (UK), Italy, Germany, France, and Spain are the five countries that had the highest number of cases, 60, 8, 5, 4, and 3 cases, respectively.

**Fig. 2:**
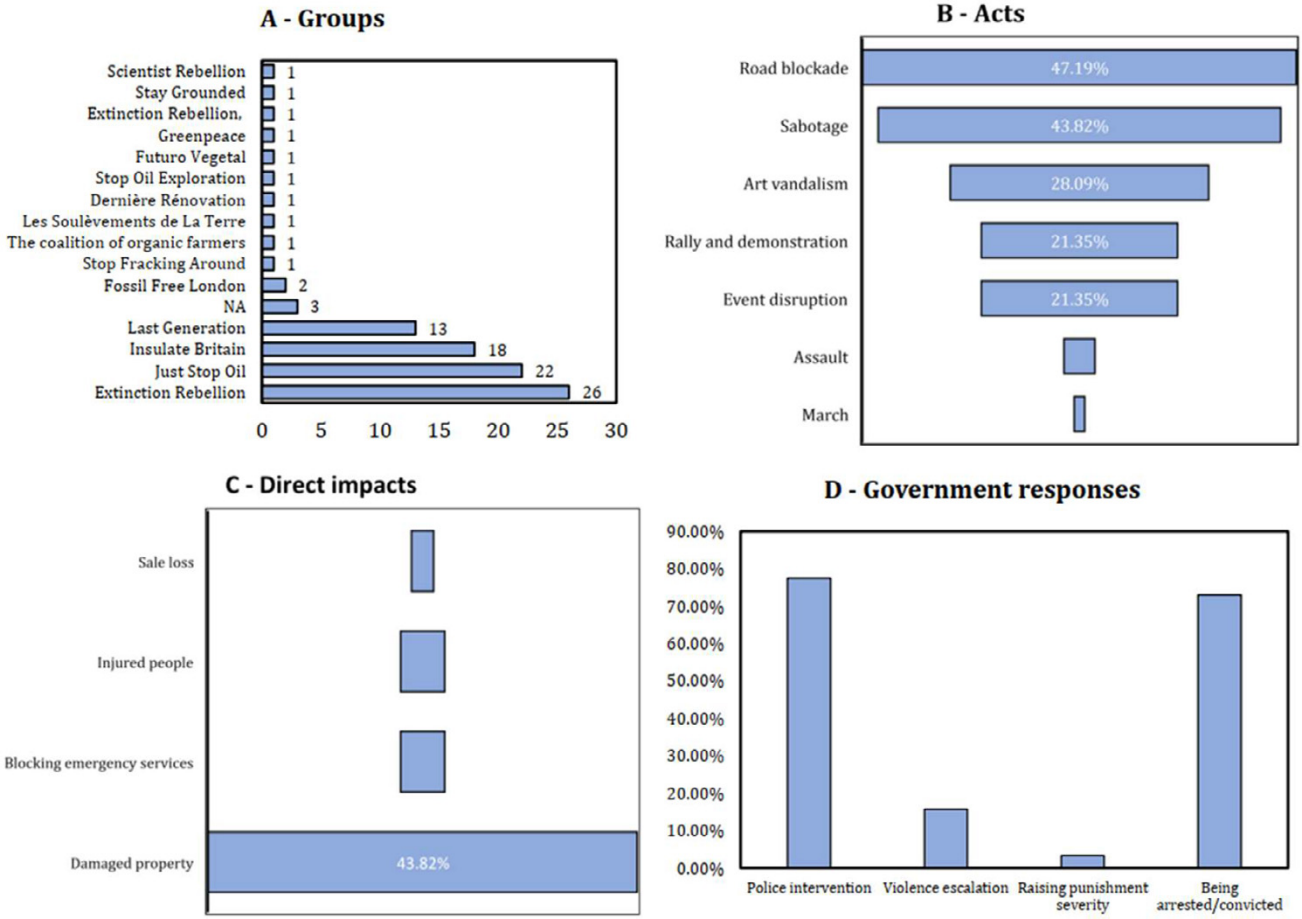
Some statistical details of blockade, vandalism, and harassment cases.

These events were conducted mainly by 14 environmentalist groups, with Extinction Rebellion, Just Stop Oil, and Insulate Britain being the three most active groups. These groups participated in 29.21%, 24.72%, and 20.22% of cases, respectively (see Fig. 2-A). Road blockade, sabotage, and art vandalism are the three most frequently conducted acts, with 47.19%, 43.82%, and 28.09% of the cases (see Fig. 2-B). The most incurred direct impact is damaged property while blocking emergency services, and injured people ranked second simultaneously, with 4.49% of the cases (see Fig. 2-C). Most blockade, vandalism, and harassment events end with the intervention of police (77.53%) and the activists being arrested or convicted (73.03%) (see Fig. 2-D).

### 3.2 Data coding

This section displays how the data in three main categories of the dataset were coded:

1) Event
2) Activists
3) Consequences

As the data were retrieved from the news, so most of the variables are categorical variables (including binary variables). We describe categorical variables using seven kinds of information corresponding with seven columns: “Sub-category,” “Variable,” “Name,” “Explanation,” “Code,” “Frequency,” and “Proportion.”

#### 3.2.1 Event

The first category of the dataset includes five sub-groups that focus on demonstrating the blockade, vandalism, and harassment events that happened: Overview of the event (four variables), targeted sector (seven variables from *A1-A7*), targeted location (two variables from *B1-B2*), targeted subjects (two variables from *C1-C2*), and affected social groups in the events (four variables from *D1-D4*) (Table 1).

**Table 1:** Description of variables in the Event category.

Sub-category	Variable	Name	Explanation	Code	Frequ- ency	Propor- tion
**Overview**	Date	Date	The date the action took place	NA	NA	NA
	Country	Country	The country the action took place	NA	NA	NA
	Location	Location of the activity	The location where the action took place	NA	NA	NA
	Groups	Environme -ntalist groups	The environmentalist groups that participated in the action	NA	NA	NA
**Targeted sector**	A1	Sport	Whether the action took place at the sports events	1 = Yes	8	8.99%
				0 = No	81	91.01%
	A2	Transportation	Whether the action took place at the conveyance of traffic or at the place associated with transportation	1 = Yes	39	43.82%
				0 = No	50	56.18%
	A3	Agriculture	Whether the action took place at agriculture-related facilities or organizations	1 = Yes	1	1.12%
				0 = No	88	98.88%
	A4	Energy	Whether the action took place at the energy-related facilities or organizations	1 = Yes	6	6.74%
				0 = No	83	93.26%
	A5	Finance	Whether the action took place at the finance-related facilities or organizations	1 = Yes	5	5.62%
				0 = No	84	94.38%
	A6	Art, science, and culture	Whether the action took place at the facilities or organizations that are related to art, science, and culture	1 = Yes	35	39.33%
				0 = No	54	60.67%
	A7	Press	Whether the action took place at the press-related facilities or organizations	1 = Yes	2	2.25%
				0 = No	87	97.75%
**Targeted location**	B1	Private/ corporate place	Whether the action happened in a private/corporate place	1 = Yes	25	28.09%
				0 = No	64	71.91%
	B2	Public place	Whether the action happened in a private/corporate place	1 = Yes	70	78.65%
				0 = No	19	21.35%
**Targeted subjects**	C1	People	Whether the action was aimed at affecting people	1 = Yes	53	59.55%
				0 = No	36	40.45%
	C2	Properties	Whether the action was aimed at affecting properties	1 = Yes	75	84.27%
				0 = No	14	15.73%
**Affected social groups**	D1	Elitist/wealthy people	Whether the action affected elitists/wealthy people	1 = Yes	12	13.48%
				0 = No	77	86.52%
	D2	Corporate shareholders	Whether the action affected corporate shareholders	1 = Yes	10	11.24%
				0 = No	79	88.76%
	D3	Politicians	Whether the action affected politicians	1 = Yes	5	5.62%
				0 = No	84	94.38%
	D4	Ordinary people	Whether the action was aimed at affecting people	1 = Yes	73	82.02%
				0 = No	16	17.98%

#### 3.2.2 Activists

The second category concentrates on factors associated with the activists, like their motivations (seven variables from *E1-E7*), fallacy and hate speech of their messages (four variables from *F1-F4*), and their act (seven variables from *G1-G7*) (Table 2).

**Table 2:** Description of variables in the Activists category.

Sub-category	Variable	Name	Explanation	Code	Freq- uency	Propor- tion
Motiva- tion	E1	Raise awareness about climate change	Whether the action was to raise public awareness about climate change	1 = Yes	75	84.27%
				0 = No	14	15.73%
	E2	Raise awareness about biodiversity loss	Whether the action was to raise public awareness about biodiversity loss	1 = Yes	8	8.99%
				0 = No	81	91.01%
	E3	Raise awareness about other environmental problems other than climate change and biodiversity loss	Whether the action was to raise public awareness about environmental problems other than climate change and biodiversity loss	1 = Yes	11	12.36%
				0 = No	78	87.64%
	E4	Raise awareness of non-environmental problems (socio-economic issues)	Whether the action was to raise public awareness about non-environmental problems (socio-economic issues)	1 = Yes	39	43.82%
				0 = No	50	56.18%
	E5	Social issues’ details	Details of the socio-economic issues	NA	NA	NA
	E6	Request/Pressure the government to act	Whether the action was to request/pressure the government to act	1 = Yes	70	78.65%
				0 = No	19	21.35%
	E7	Request/Pressure business to stop operation/emission	Whether the action was to request/pressure business to stop operation/emission	1 = Yes	15	16.85%
				0 = No	74	83.15%
Fallacy and hate speech	F1	Fallacy	The fallacy level of the messages provided by the activists	2 = Fallacy	17	19.10%
				1 = Suspicious fallacy	3	3.37%
				0 = No fallacy	60	67.42%
	F2_1	Fallacy evidence	Evidence for variable F1	NA	NA	NA
	F2_2	Fallacy types	Types of fallacy	NA	NA	NA
	F3	Hate speech	The level of hate speech in the messages provided by the activists	2 = Hate speech	3	3.37%
				1 = Suspicious hate speech	3	3.37%
				0 = No hate speech	72	80.90%
	F4	Hate speech evidence	Evidence for variable F3	NA	NA	NA
Act	G1	Rally and demonstration	Whether the action is conducted in the form of a rally or demonstration	1 = Yes	19	21.35%
				0 = No	70	78.65%
	G2	March	Whether the action is conducted in the form of a march	1 = Yes	1	1.12%
				0 = No	88	98.88%
	G3	Event disruption	Whether the action conducted disrupts the event or activities that are happening	1 = Yes	19	21.35%
				0 = No	70	78.65%
	G4	Sabotage	Whether the action conducted to sabotage the targeted properties	1 = Yes	39	43.82%
				0 = No	50	56.18%
	G5	Road blockade	Whether the action conducted to block the road	1 = Yes	42	47.19%
				0 = No	47	52.81%
	G6	Assault	Whether the action conducted aims to assault or harass a targeted person/people	1 = Yes	3	3.37%
				0 = No	86	96.63%
	G7	Art vandalism	Whether the action conducted under the form of art vandalism	1 = Yes	25	28.09%
				0 = No	64	71.91%

In this category, variable F1 reflects whether the message provided by the activists when they conducted the action is fallacious. Such messages were retrieved from the news regarding the event. To identify whether the message is fallacious, we assessed if the message's argument falls into the following 11 common types of fallacies [13], [14], [15]:

1. *Ignoratio elenchi* (“ignoring refutation”): an informal fallacy of demonstrating an argument that may or may not be logically valid and sound but is not related to the issue in question.
2. *Argumentum ad hominem* (“argument against the person”): a fallacious strategy that puts the questioner but not the argument under scrutiny.
3. *Petitio principii* (“begging the question”): an informal fallacy that assumes the truth of a premise that is supposed to be proven in the argument.
4. *Non sequitur* (“it does not follow”): an argument provides evidence but does not really prove the point.
5. *Post hoc ergo propter hoc* (“after this, therefore because of this”): an informal fallacy that considers one event to be the cause of a later event because it occurred earlier.
6. *Argumentum ad populum* (“appeal to the people”): a fallacy that considers the claim as true based on its popularity.
7. False dilemma (or false dichotomy): an informal fallacy based on the premise that erroneously limits the available options.
8. Card stacking (or cherry picking): a fallacy that uses incomplete evidence (i.e., individual cases or data that seem to confirm a particular position) and ignores all other important evidence.
9. False equivalence (or false analogy): an informal fallacy in which the differences between two compared things outweigh the similarities, or consider that if two things are alike in one or a few ways, they are alike in all ways.
10. Hasty generalization: an informal fallacy in which a conclusion is drawn quickly from inadequate evidence.
11. Association fallacy: a formal logical fallacy that asserts the property of one thing must also be properties of another thing.

If the messages of the activists fall into any of the above categories, the observation will be coded as ‘fallacy’. However, identifying fallacies in common debates is challenging because they are often entrenched in rhetorical patterns that conceal the logical relationships between propositions. Therefore, for any messages that we could not identify as ‘fallacy’ or ‘no fallacy’, they are referred to as ‘suspicious fallacy’. The evidence of the fallacy (i.e., messages provided by the activists) is shown in the column of variable *F2_1*, while the types of the fallacy of the messages (based on 11 common types of fallacies above) are shown in the column of variable *F2_2*.

To identify hate speech in the activists’ messages, we followed Howard [16] to adopt the three-pronged characterization of hate speech offered by Parekh [17]. If any identified message meets all the following criteria, it will considered hate speech:

1. It “is directed against a specified or easily identifiable individual or, more commonly, a group of individuals based on an arbitrary or normatively irrelevant feature”.
2. It “stigmatizes the target group by implicitly or explicitly ascribing to it qualities widely regarded as undesirable”.
3. It casts the “target group…as an undesirable presence and a legitimate object of hostility.”

Variable *F3* is used to demonstrate whether the provided message is ‘hate speech’, ‘suspicious hate speech’ (when we are unsure if the message meets all the criteria to be considered hate speech), or ‘no hate speech’, while the column of variable *F4* displays the evidence of the hate speech (i.e., messages provided by the activists).

To record the types of activism conducted by the activists, we based on the news's description of the event. In general, we identified seven main types of activism reported by the news: rally and demonstration (indicated by variable *G1*), march (indicated by variable *G2*), event disruption (indicated by variable *G3*), sabotage (indicated by variable *G4*), road blockade (indicated by variable *G5*), assault (indicated by variable *G6*), and art vandalism (indicated by variable *G7*). It should be noted that we considered the activism event as a series of activities, so multiple types of activism can be conducted in a single event. For example, the event conducted by Just Stop Oil campaigners on April 28th, 2022, at the petrol pumps on the M25 motorway is considered to be simultaneously disrupting the occurring activities at the petrol pumps (indicated by variable *G3*), sabotaging the properties at the station (indicated by variable *G4*), and blocking the road leading to the station (indicated by variable *G5*).

#### 3.2.3 Consequences

The final category comprises the data demonstrating the direct impacts of the blockade, vandalism, and harassment events (five variables from *H1-H5*) and the responses of the government (four variables from *I1-I4*) (Table 3).

**Table 3:** Description of variables in the Consequences category.

Sub-category	Variable	Name	Explanation	Code	Frequ- ency	Propo- rtion
**Direct impacts**	H1	Damaged property	Whether the action damaged property	1 = Yes	39	43.82%
				0 = No	50	56.18%
	H2	Sale loss	Whether the action caused a sales loss	1 = Yes	2	2.25%
				0 = No	87	97.75%
	H3	The total amount of loss due to the action	The total amount of loss due to the action	NA	NA	NA
	H4	Injured people	Whether there were injured people due to the action	1 = Yes	4	4.49%
				0 = No	85	95.51%
	H5	Blocking emergency services	Whether the action blocked emergency services	1 = Yes	4	4.49%
				0 = No	85	95.51%
**Govern- ment**	I1	Police involvement	Whether there was police involvement when the event was happening	1 = Yes	69	77.53%
				0 = No	20	22.47%
	I2	Violence escalation	Whether there was violence escalation between the protestors, policemen, and other citizens	1 = Yes	14	15.73%
				0 = No	75	84.27%
	I3	Raising punishment severity	Whether the government raised the bar of punishment	1 = Yes	3	3.37%
				0 = No	86	96.63%
	I4	Being arrested or convicted	Whether the people were convicted/arrested due to the action	1 = Yes	65	73.03%
				0 = No	24	26.97%

When reading the news regarding the blockade, vandalism, and harassment events, we identified four kinds of potential adverse direct impacts caused by the events: damaging the property (e.g., burning cars, painting a private jet, etc.), causing sale loss to a business(es) that was operating in the area, injuring people (e.g., blocking newspaper daily delivery), and blocking emergency services (e.g., ambulance, etc.). These impacts are represented by variables *H1, H2, H4*, and *H5*, respectively. If any of these consequences are reported on the news, they will be coded as ‘1’, representing ‘yes’; otherwise, ‘0’, representing ‘no’.

In the news regarding the blockade, vandalism, and harassment events, the responses from the government were often reported. The intensity of the response ranges from none to arrestment and conviction. To record such responses, we created four variables, from *I1* to *I4*. If the news reports the involvement of the police at the scene by either protecting the public itself, resolving the disruption, blockade, and vandalism, or making the arrestment, it will be coded as ‘1’, representing ‘yes’ in column of variable *I1*; otherwise, it will be coded as ‘0’, representing ‘no’. Whether the news reports that the government decides to raise the bar of punishment rapidly after the event (e.g., imposing more severe fines or possible jail terms, etc.) is reflected through variable *I3*. Meanwhile, whether the news reports the activists are arrested or convicted after conducting the event is shown by variable *I4*.

In some cases, the police or people affected by the activism activities used force (e.g., clashing, deliberately driving into activists) to stop the events or break the blockade. If the news reports any actions using physical force done by the police or other people at the scene to stop the events, they will be coded as ‘1’, representing ‘yes’ in the column of variable *I2*; otherwise, they will be coded as ‘0’, representing ‘no’.

## 4 Experimental Design, Materials and Methods

### 4.1 Data design and collection procedure

The dataset was generated with two main stages: 1) identifying the event and 2) recording the metadata of the event.

#### 4.1.1 Event identification stage

To effectively identify blockade, vandalism, and harassment events carried out by environmental activist groups, we employed a thorough approach using a variety of data collection methods from public information sources, social media, and press releases. The process involves four steps.

**Step 1:** Defining the scope and keywords

In the first step, we carefully selected keywords to cover a broad spectrum of activities and possible incidents related to environmental activism. This ranged from general terms that encapsulate common forms of activism to more specific terms that detail different types of events or actions. We organized keywords according to specific organizations for targeted searches concerning known activist groups and issue-specific keywords to concentrate on protests against particular environmental issues. Additionally, we utilized location and date-specific keywords to refine our searches to specific areas or time periods of interest, thereby enhancing the relevance of our searches.

The initial groups of keywords were extracted from articles covering prominent events that were trending in the media. We evaluated and refined our keywords based on new articles, preparing them for the subsequent round of searches. This process also included the generation of new keywords, supported by tools like ChatGPT, which leveraged the information collected from previously collected articles.

Keyword groups include (see Table 4):

- Basic keywords: Encompassing terms related to common types of environmentally-related activities, protests driven by environmental concerns, and climate change activism.
- Action-specific keywords: Describing events where activists block access to areas or buildings to draw attention to their cause, where property is damaged or distorted during environmental protests, or where individuals or organizations targeted by activists face threats or pressure.
- Representative group keywords: The name of specific environmental activist groups known to conduct such events.
- Environmental issue-specific keywords: Relating to specific environmental issues being protested, helping to narrow events down to those most relevant to the research.

**Table 4:** Keyword groups.

Keyword groups	Keywords
Basic keywords	1. Climate protest 2. Climate activist 3. Environmental activism 4. Climate change demonstration 5. Eco-protest 6. Eco-activism 7. Green activism 8. Sustainable protest 9. Environmental justice rally 10. Climate emergency action
Action-specific keywords	1. Road blockade 2. Infrastructure vandalism 3. Construction site occupation 4. Fire setting in protest 5. Wall building as blockade 6. Art attack protest 7. Luxury asset targeting (yachts, private jets) 8. Public disruption protest 9. Flashbang and teargas response 10. Legal protest challenge 11. Disruptive road protests 12. Glue protests at art exhibitions
Representative group keywords	1. Extinction Rebellion (XR) 2. Letzte Generation 3. Greenpeace 4. Stop Fracking Around 5. Earth Uprisings (Les Soulèvements de la Terre) 6. Just Stop Oil 7. Ultima Generazione 8. Climate Defenders 9. Money Rebellion 10. Last Generation
Environmental issue-specific keywords	1. Fossil fuel exploration protest 2. Motorway project opposition 3. Reservoir project protest 4. Agricultural land preservation 5. Anti-coal mining demonstration 6. Oil pipeline resistance 7. Deforestation activism 8. Biodiversity loss protest 9. Air pollution campaign 10. Water resource protection 11. Global warming rally 12. Plastic pollution fight

For a targeted search, we combine keywords from different keyword groups in one query. For example: “climate activist”, “luxury asset targeting”, “yachts”.

**Step 2:** Collecting data through various channels

To identify relevant events, we utilized three primary channels guided by our predefined keywords:

­ Public media:
  ○ We used Google search tools to collect news articles associated with our keywords.
­ Social media:
  ○ Monitoring tools: Utilizing tools like Brandwatch to monitor mentions across platforms such as Twitter, Facebook, and Instagram related to environmental activist groups and disruptive keywords.
  ○ Hashtag tracking: Tracking relevant hashtags on social media platforms.
­ Press releases and reports:
  ○ Official websites: Checking official websites of activist groups for press releases.
  ○ Government and law enforcement announcements: These may provide official information on incidents related to blockade, vandalism, and harassment.

We stopped collecting new data when it reached the saturation point: the newly found information became similar to those events that we had found.

**Step 3:** Organizing and storing event data

We established a database to store and categorize the collected data systematically. This database included fields for the date, location, type of event, related groups, a detailed description, and the source of information. With each new search round, we updated the database with fresh data and enriched existing entries with supplementary details.

**Step 4:** Verifying the event data

Ensuring the reliability and legal compliance of information sources, especially when collecting data on sensitive topics like environmental activism. Our approach to ensuring source credibility included several strategies:

­ Evaluating source credibility: We sought out sources written by recognized experts or authoritative entities within the environmental research domain.
­ Affiliation: We considered the affiliations of our sources, with reputable news outlets typically being viewed as reliable.
­ Citations: We checked whether other reputable sources cited the source, indicating its reliability and acceptance within the field.
­ Assessing source objectivity: We checked the potential biases of the sources to help critically evaluate the information provided.
­ Cross-referencing information: We verified the information about events by cross-referencing it with multiple reliable sources. Consistency across different sources often signals reliability.
­ Fact-checking websites: For quick verification of claims related to environmental issues and activism, we also utilized fact-checking websites.

#### 4.1.2 Metadata retrieval stage

In the second step, we designed a questionnaire to retrieve the metadata from the collected information sources (primarily news from reputable news outlets due to their credibility and the thoroughness of the news itself) for each event. The Mindsponge Theory was employed to design the structure of the questionnaire. The theory is a theory of mind developed from the mindsponge mechanism, a socio-psychological framework, and recent evidence from brain and life sciences [18,19]. Specifically, the Mindsponge Theory considers the mind and the environment as two major spectrums. The mind is defined as an information collection-cum-processor, while the environment is theoretically a larger information-processing system (e.g., the Earth system, the social system, etc.) that includes the human mind.

Based on this categorization, we deem the activist conducting the blockade, vandalism, and harassment events as minds, while the backgrounds where the events took place are deemed as the surrounding environment with which the activist interacted. Therefore, Events and Activists are classified as two primary categories of the dataset. Moreover, the interactions between the activists and the surrounding environment eventually led to certain results. Such results are classified into the third category of the dataset: Consequences.

Detailed descriptions of each variable and how the variable was generated are shown in the codebook deposited online and in the Tables shown in Section 2.2.

Two authors implemented the event identification and metadata retrieval processes from September 8th to September 26th, 2023. The two authors also crosschecked to ensure the quality of the data retrieval and discussed with each other when encountering any ambiguous information. Eventually, 89 cases were recorded. The metadata's primary sources (i.e., links to the news) are included in the last column of the dataset.

### 4.2 Dataset validation

The Bayesian Mindsponge Framework (BMF) analytics was employed to check the validity of the dataset [20,21]. The method employs the Mindsponge Theory for theoretical reasoning and Bayesian inference for statistical analysis [18,^22^,23], which is also compatible with the dataset's design.

To check the validity of the dataset, we conducted an analysis to examine which types of activism are associated with a higher probability of escalation into violence. The Mindsponge Theory suggests that individuals’ thinking and behaviors are products of the information process of the mind (the information collection-cum-processor), which aims to maximize the perceived benefits and minimize the perceived costs for prolonging the existence of the system in one way or another, such as through survival, growth, and reproduction [18,24]. Based on this reasoning, we assume that a violent reaction is a costly action that can cause detrimental effects to all the people involved. Therefore, the situation will escalate into violence when at least one party is involved in or affected by the activism events when they perceive violence to be more beneficial rationally and emotionally than non-violence alternatives. If the estimated results align with this theoretical reasoning, the data quality can be deemed validated by the Mindsponge Theory.

The analysis was conducted using the bayesvl R package to estimate the following model [25]:

(1.1) $$ViolenceEscalation\;∼\;normal\left(\log\left(\frac{\mu_{i}}{1-\mu_{i}}\right),\sigma \right)$$

(1.2) $$\begin{array}{ccc} \log\left(\frac{\mu_{i}}{1-\mu_{i}}\right) & = & \beta_{0}+\beta_{1\;}*RallyDemonstration_{i}+\beta_{2}*March_{i}+\beta_{3}*EventDisruption_{i}\\ & & +\beta_{4}*Sabotage_{i}+\beta_{5}*RoadBlockage_{i}+\beta_{6}*Assault_{i}\\ & & +\beta_{7}*ArtVandalism_{i} \end{array}$$

(1.3) β∼normal(M,S)

The probability around the mean $\log(\frac{\mu_{i}}{1-\mu_{i}})$ is determined by the form of the normal distribution, whose width is specified by the standard deviation σ. $\mu_{i}$ indicates the event i’s probability of being escalated into violence; $RallyDemonstration_{i}$ indicates whether event i was conducted in the form of a rally and demonstration; $March_{i}$ indicates whether event i was conducted in the form of a march; $EventDisruption_{i}$ indicates whether event i was conducted in the form of event disruption; $Sabotage_{i}$ indicates whether event i was conducted in the form of sabotage; $RoadBlockage_{i}$ indicates whether event i was conducted in the form of road blockade; $Assault_{i}$ indicates whether event i was conducted in the form of assault or harassment; $ArtVandalism_{i}$ indicates whether event i was conducted in the form of art vandalism. Model 1 has nine parameters: the coefficients, $\beta_{1\;}$ - $\beta_{7\;}$, the intercept, $\beta_{0}$, and the standard deviation of the “noise”, σ. The coefficients of the predictor variables are distributed as a normal distribution around the mean denoted M and with the standard deviation denoted S.

**Fig. 3:**
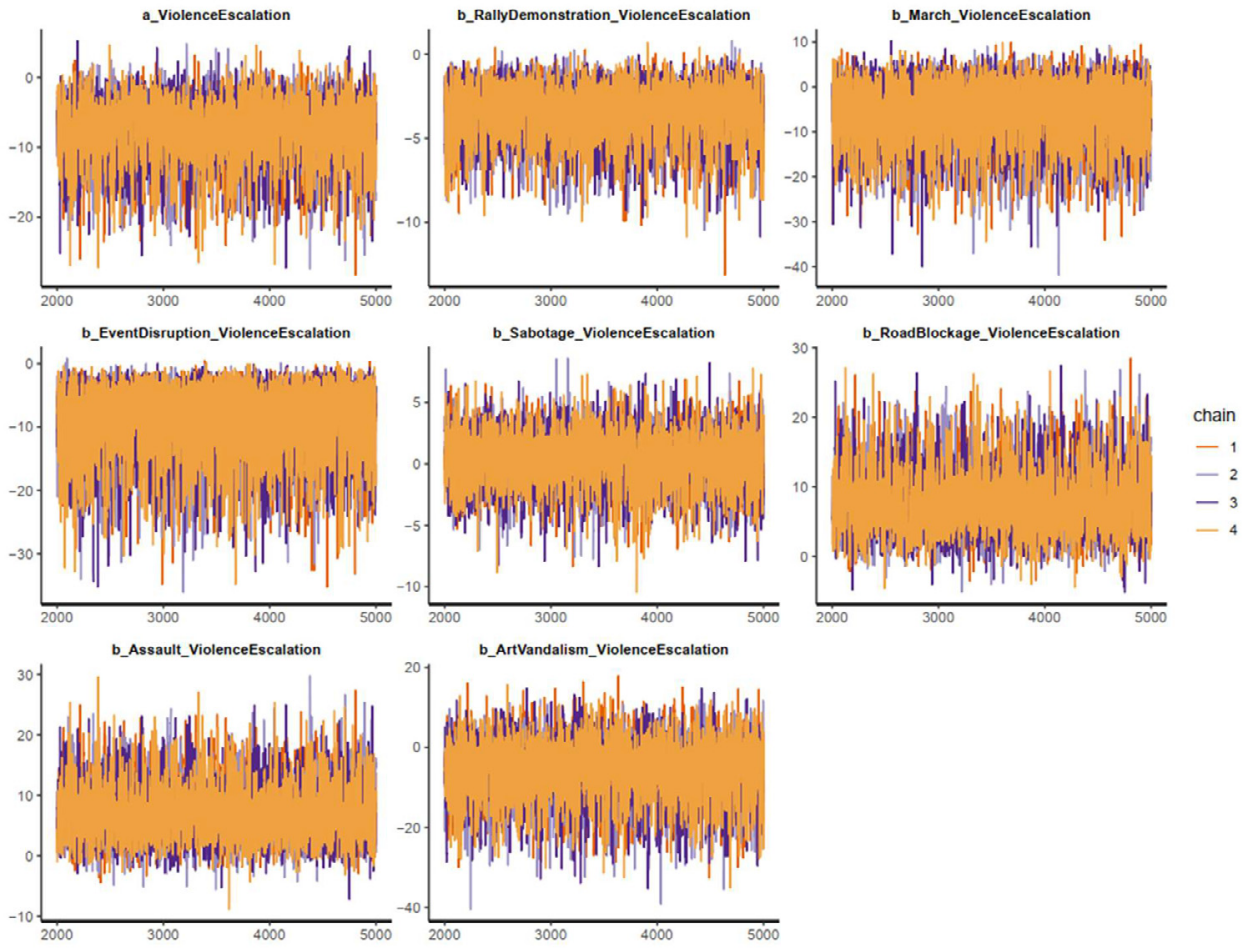
Model 1's trace plots.

All the estimated results of Model 1 are shown in Table 5. The effective sample size (*n_eff*) is larger than 1000, and the shrink factor (*Rhat*) is equal to 1 in all cases of parameters. These statistics suggest that Markov chains of Model 1 are all well-convergent. Visually, the Markov chains shown in the trace plots also confirm the convergence: fluctuating around a central equilibrium (see Fig. 3). The estimated results are qualified for interpretation as the Markov chains are convergent.

**Table 5:** Estimated results of Model 1.

Parameters	Mean	Standard deviation	*n_eff*	*Rhat*
*Constant*	−8.10	4.61	3800	1
*RallyDemonstration*	−3.52	1.55	5832	1
*March*	−5.86	6.85	5784	1
*EventDisruption*	−9.69	5.69	4682	1
*Sabotage*	0.38	2.11	4568	1
*RoadBlockage*	8.16	4.46	3208	1
*Assault*	7.56	4.64	3230	1
*ArtVandalism*	−5.98	7.21	5152	1

**Fig. 4:**
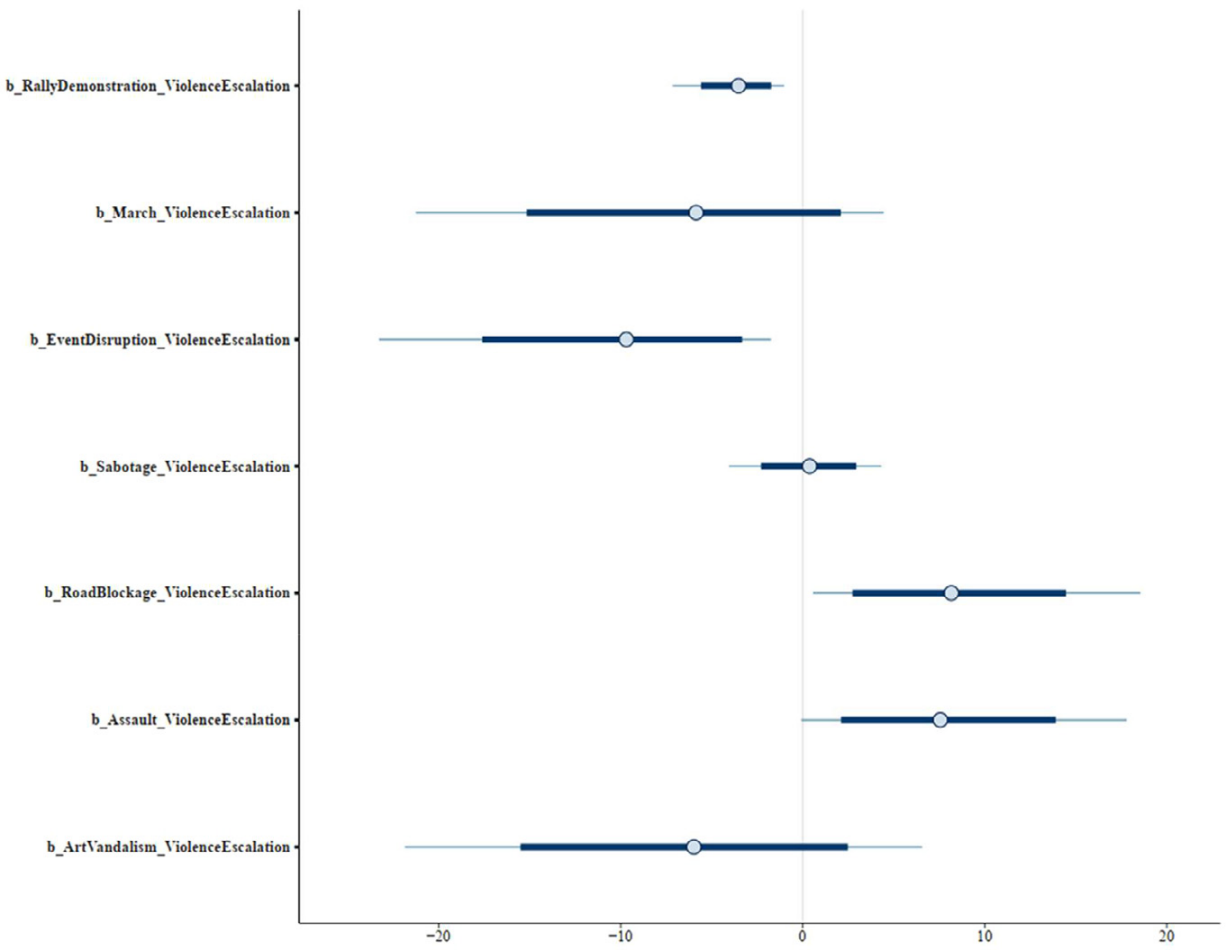
Model 1's posterior distributions.

The estimated results show that rally and demonstration ($M_{RallyDemonstration}$= −3.52 and $S_{RallyDemonstration}$= 1.55), march ($M_{March}$= −5.86 and $S_{March}$= 6.85), event disruption ($M_{EventDisruption}$= −9.69 and $S_{EventDisruption}$= 5.69), and art vandalism ($M_{ArtVandalism}$= −5.98 and $S_{ArtVandalism}$= 7.21) are negatively associated with the probability of violence escalation. Meanwhile, road blockade ($M_{RoadBlockage}$= 8.16 and $S_{RoadBlockage}$= 4.46) and assault ($M_{Assault}$= 7.56 and $S_{Assault}$= 4.64) are positively associated with the probability of violence escalation, and sabotage has an ambiguous effect. The coefficients’ posterior distributions are shown in Fig. 4.

These results make sense as rallies, demonstrations, and marches are two common types of activism and legally accepted in high-income Western countries, while event disruption and art vandalism are two types of activism that aim at entertainment purposes and seemingly do not have negative effects on other people's survival demand. However, road blockade can cause adverse effects on people using the roads (e.g., traffic safety, delayed work, emergency, etc.), while assault or harassment directly threatens the person's safety. Therefore, the dataset can be considered valid through the theoretical reasoning of the Mindsponge Theory.

The R code snippet used for analyzing the data is shown in Box 1.

Box 1 **R code snippet for data analysis**

**# Load data and package** data1<-read.csv(“D:/…/Environment art_Dataset_sorted.csv”,header = TRUE,stringsAsFactors = TRUE) library(bayesvl)

**# Prepare data** data1$RallyDemonstration<-data1$G1 data1$March<-data1$G2 data1$EventDisruption<-data1$G3 data1$Sabotage<-data1$G4 data1$RoadBlockage<-data1$G5 data1$ArtVandalism<-data1$G7

data1$ViolenceEscalation<-data1$I2

**# Model construction** model1a<-bayesvl() model1a<-bvl_addNode(model1a,“ViolenceEscalation”,“binom”) model1a<-bvl_addNode(model1a,“RallyDemonstration”,“binom”) model1a<-bvl_addNode(model1a,“March”,“binom”) model1a<-bvl_addNode(model1a,“EventDisruption”,“binom”) model1a<-bvl_addNode(model1a,“Sabotage”,“binom”) model1a<-bvl_addNode(model1a,“RoadBlockage”,“binom”) model1a<-bvl_addNode(model1a,“Assault”,“binom”) model1a<-bvl_addNode(model1a,“ArtVandalism”,“binom”)

model1a<-bvl_addArc(model1a,“RallyDemonstration”,“ViolenceEscalation”,“slope”) model1a<-bvl_addArc(model1a,“March”,“ViolenceEscalation”,“slope”) model1a<-bvl_addArc(model1a,“EventDisruption”,“ViolenceEscalation”,“slope”) model1a<-bvl_addArc(model1a,“Sabotage”,“ViolenceEscalation”,“slope”) model1a<-bvl_addArc(model1a,“RoadBlockage”,“ViolenceEscalation”,“slope”) model1a<-bvl_addArc(model1a,“Assault”,“ViolenceEscalation”,“slope”) model1a<-bvl_addArc(model1a,“ArtVandalism”,“ViolenceEscalation”,“slope”)

**# Generate Stan code** model_string1a<- bvl_model2Stan(model1a) cat(model_string1a)

**# Model Fit** model1a<-bvl_modelFit(model1a, data1, warmup = 2000, iter = 5000, chains = 4,cores = 4)

**# Visualize logical network of Model 1** bvl_bnPlot(model1a)

**# Visualize trace plots of Model 1** bvl_plotTrace(model1a)

**# Visualize posterior distributions of Model 1** bvl_plotIntervals(model1a,c(“b_RallyDemonstration_ViolenceEscalation”,“b_March_ViolenceEscalation”,“b_EventDisruption_ViolenceEscalation”,“b_Sabotage_ViolenceEscalation”,“b_RoadBl-ockage_ViolenceEscalation”,“b_Assault_ViolenceEscalation”,“b_ArtVandalism_Violence- Escalation”))

Alt-text: Unlabelled box

## Limitations

The method of identifying the blockage and vandalism events based on manual search is not exhaustive, so the dataset can only cover the events that were popular at the time of identification. Moreover, the metadata were mainly retrieved from news, so they face the risk of not being complete. For example, data demonstrating the sales loss and amount of loss caused by the activities are not complete because they are difficult to estimate, and not all losses caused by the events are reported on the news. The language used for search queries was English, so the geographical bias of the data is acknowledged (e.g., most events are in Western countries). Therefore, this dataset can be a valuable resource for conducting preliminary analysis, but the results should not be generalized. Future data collection should be conducted through on-the-scene observations or reports from the activists and police officers to validate the current dataset.

## Ethics Statement

The authors have read and followed the ethical requirements for publication in Data in Brief and confirmed that the current work does not involve human subjects, animal experiments, or any data collected from social media platforms.

## CRediT authorship contribution statement

**Quan-Hoang Vuong:** Conceptualization, Writing – original draft, Methodology. **Minh-Hoang Nguyen:** Conceptualization, Data curation, Writing – original draft, Writing – review & editing, Visualization, Investigation. **Viet-Phuong La:** Software, Validation, Investigation.

## Data Availability

Blockage, vandalism, and harassment activities for the cause of climate change mitigation: data deposit (Original data) (Zenodo).

## Appendix Supplementary materials

Image, application 1
